# Potential heterogeneity of epcoritamab associated adverse events in demographics and dosing regimens

**DOI:** 10.3389/fmed.2026.1832550

**Published:** 2026-07-23

**Authors:** Zhongming Yu, Cheng Jiang, Wenfei Zhou, Jingrui Jin, Zhilu Chen

**Affiliations:** 1 Zhejiang Academy of Traditional Chinese Medicine, Tongde Hospital of Zhejiang Province, Hangzhou, Zhejiang, China; 2 Zhejiang Provincial Key Laboratory of Disease-Syndrome Integrated Cancer Prevention and Treatment, Tongde Hospital of Zhejiang Province, Hangzhou, Zhejiang, China; 3 Zhejiang Provincial Key Laboratory of Traditional Chinese Medicine for Pharmacodynamic Material Basis Research of Chinese Medicine, Tongde Hospital of Zhejiang Province Affiliated to Zhejiang Chinese Medical University, Hangzhou, Zhejiang, China

**Keywords:** cytokine release syndrome, epcoritamab, FAERS, immune effector cell-associated neurotoxicity syndrome, JADER

## Abstract

**Background:**

Epcoritamab shows potentially curable efficacy in relapsed or refractory B-cell non-Hodgkin’s lymphoma, but potential heterogeneity in its adverse events (AEs) across demographics and dosing regimens remain unexplored.

**Methods:**

This study comprehensively analyzed potential heterogeneity of epcoritamab associated AEs using the data from the FDA Adverse Event Reporting System (FAERS) database and supplemented it with data from the Japanese Adverse Drug Event Report (JADER) database. Based on the clinical characteristics of the epcoritamab-associated AE reports, the potential heterogeneity of AEs across demographic and dosing regimen subgroups was investigated via reporting odds ratio algorithm and Fisher’s exact test with Bonferroni correction. Subsequently, sensitivity analysis was performed to validate the potential variations.

**Results:**

Patients receiving 0.16 mg dose reported significantly higher frequency of cytokine release syndrome (CRS), especially within the first four treatment weeks. Immune effector cell-associated neurotoxicity syndrome (ICANS) was reported with a significantly higher frequency among patients over the age of 65 compared to those aged 18–65.

**Conclusion:**

This study revealed the potential heterogeneity of epcoritamab associated AEs in demographics and dosing regimens. Enhanced CRS monitoring should be prioritized during the initial administration of epcoritamab treatment. Monitoring ICANS may be of particular importance in older patients. Further research is warranted to elucidate the case causality of these associations.

## Highlights

- Cytokine Release Syndrome: Enhanced monitoring should be prioritized during the initial administration.
- Immune effector cell-associated neurotoxicity syndrome: Monitoring may be of particular importance in older patients.

## 1 Introduction

Non-Hodgkin’s lymphoma (NHL) is the most prevalent hematological malignancy globally (1), with approximately 85–90% of cases originating from B cells (2). B-cell NHL comprises both aggressive and indolent types (3–5). Among them, diffuse large B cell lymphoma (DLBCL) is recognized as the most common subtype of aggressive lymphoma (6, 7), with approximately 30–40% of affected individuals experiencing relapsed or refractory disease and poor prognosis, characterized by a median survival duration of merely 5–7 months (8). Indolent B-cell NHL, such as follicular lymphoma and chronic lymphocytic leukemia, typically exhibit a response to therapy but are rarely curable, and patients who experience an early relapse tend to have unfavorable prognoses (9–11). Patients with relapsed or refractory B-cell NHL face a restricted range of treatment options (12, 13). Limited response rates secondary to lack of significant T-cell infiltration in the tumor site is a major problem.

T-cell redirecting immunotherapeutic approaches including bispecific T cell engager therapy (BiTE) have the potential to revolutionize B-cell NHL therapy (14). It is well established that CD20 is widely expressed on malignant B cells (15). Epcoritamab is an antibody utilized in BiTE, targeting CD3 and CD20 that redirects and activates T cells to kill CD20-expressing malignant cells (16). On May 19, 2023, the United States Food and Drug Administration (FDA) granted approval to epcoritamab for the treatment of adult patients with relapsed or refractory DLBCL, including DLBCL from indolent lymphoma, and high-grade B-cell lymphoma after two or more lines of systemic therapy (17). The current clinical development of epcoritamab encompasses a prospective indication for the treatment of relapsed or refractory follicular lymphoma and chronic lymphocytic leukemia (18, 19). The clinical development of epcoritamab, both as a standalone treatment and in combination with standard of care therapies for mature B-cell NHLs, is ongoing worldwide (20). Research has shown that subcutaneous administration of epcoritamab can lead to significant and potentially curable responses for patients with relapsed or refractory B-cell NHL^16^.

Several adverse events (AEs) associated with epcoritamab have been reported, such as cytokine release syndrome (CRS), Immune effector cell-associated neurotoxicity syndrome (ICANS), neutropenia, pyrexia, fatigue, nausea, and diarrhea, among others (21). A recent study has explored AE signals related to epcoritamab using data from the FDA Adverse Event Reporting System (FAERS) (22), the world’s largest pharmacovigilance database for detecting drug-related AE signals (23–25), identifying a new AE (hydronephrosis) with epcoritamab. However, the primary focus of current studies based on spontaneous reporting database has been on identifying novel AE signals. Till now, the potential heterogeneity in demographics and dosing regimens of epcoritamab associated-AEs remain largely unexplored.

This study analyzed the potential heterogeneity of epcoritamab associated AEs across demographic and dosing regimen subgroups using the data from the FAERS database and supplemented it with data from the Japanese Adverse Drug Event Report (JADER) database. The findings offer insights to explore potential differences in epcoritamab associated AEs that warrant further investigation. To the best of our knowledge, this is the first comprehensive and systematic pharmacovigilance study of epcoritamab-related AEs concerning potential subgroup differences.

## 2 Methods

### 2.1 Data source and collection

Raw data covering the period from the second quarter of 2023 (the quarter in which epcoritamab was approved by the FDA) to the second quarter of 2024 (the most recent quarter available at the start of this study) were downloaded from the FAERS database. The AE reports of epcoritamab were identified by searching for the “EPCORITAMAB” or “EPCORITAMAB-BYSP” in the “prod_ai” column. The data in the JADER database spanned the same period with those in the FAERS database to reduce the deviations in different periods. The reports of epcoritamab were identified using generic name “エプコリタマブ(遺伝子組換え)” in the “医薬品(一般名)” column and “被疑薬” in the “医薬品の関与” column. The standardized Medical Dictionary for Regulatory Activities (MedDRA) version 27.0 was used. The additional details regarding data source and collection can be found in previously published articles (26–30).

### 2.2 Statistical analysis

Using the data from FAERS and JADER databases, the AE signals of epcoritamab were explored at the System Organ Class (SOC) and Preferred Term (PT) levels using four disproportionality algorithms, including reporting odds ratio (ROR) (31, 32), proportional reporting ratio (PRR) (33, 34), Bayesian confidence propagation neural network (BCPNN) (33–35), and the multi-item gamma Poisson shrinker (MGPS) (33–35).

Based on the FAERS database, the clinical characteristics of the epcoritamab-associated AE reports were examined. The potential differences of epcoritamab associated AEs among subgroups were mined using the ROR algorithm and Fisher’s exact test. To reduce the noise caused by rare AEs and make the multiple correction more clinically meaningful, Fisher’s exact test was used in combination with Bonferroni correction, and only AEs with at least 20 cases were considered. The sensitivity analysis was conducted to further verify the potential differences. In all signal detection, subgroup analysis, and sensitivity analysis, the signals related to the SOC of “neoplasms benign, malignant and unspecified (incl cysts and polyps)” and “injury, poisoning and procedural complications,” which may result from disease progression, failure to treatment, or wrong dosing schedule, were considered as epcoritamab-unrelated signals and therefore excluded. All data processing and statistical analysis were performed using Python 3 programming language in Jupyter Notebook version 6.4.12. The additional details regarding statistical analysis can be found in Supplementary Tables S1–S4 and previously published articles (26–30).

## 3 Results

### 3.1 Signals detection

**Figure 1:**
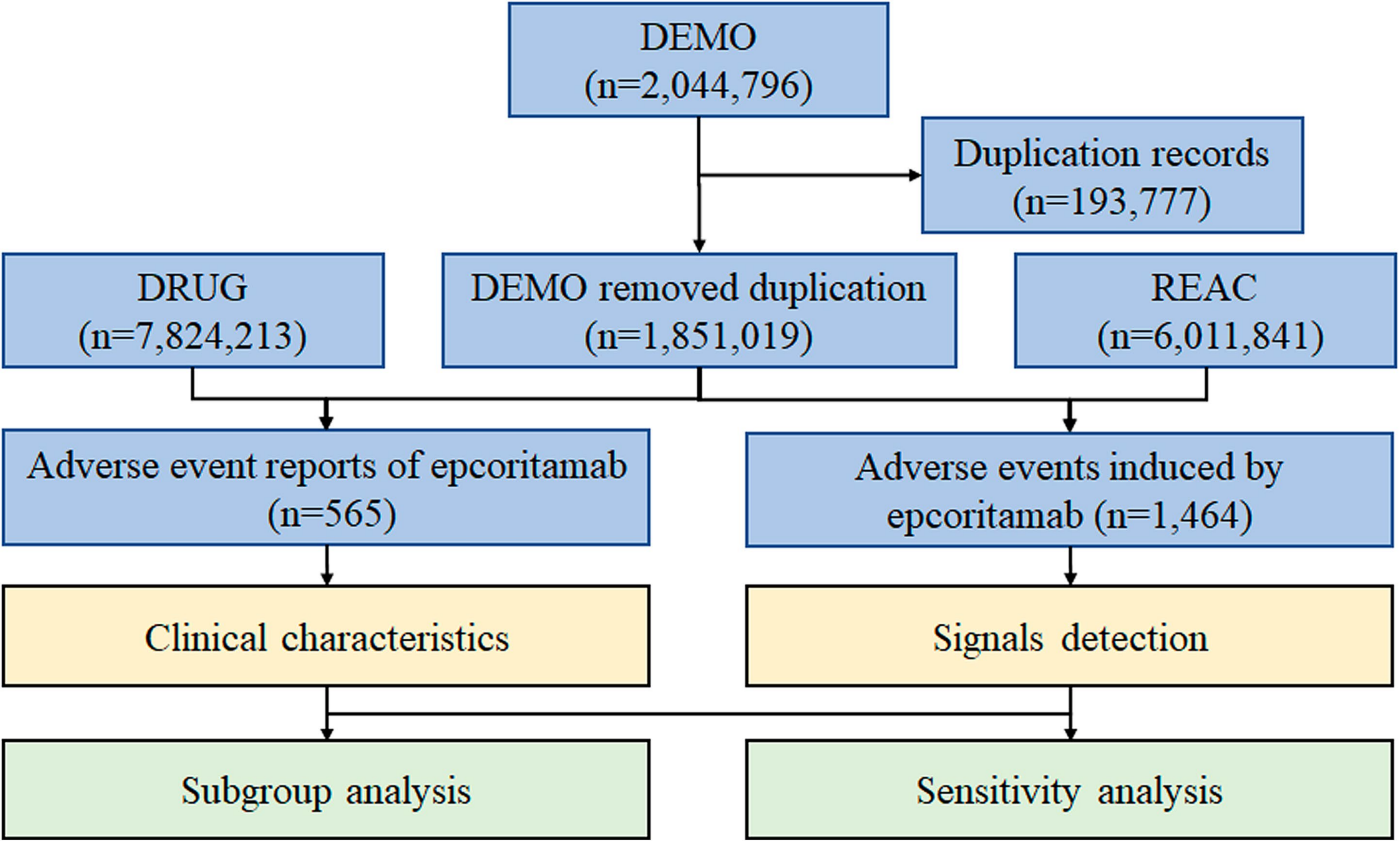
Flow diagram of data collection and analysis of epcoritamab-associated adverse events from the FAERS database. DEMO, patient demographic and administrative information; DRUG, drug information; REAC, coded for the adverse events.

A total of 565 AE reports and 1,464 AEs associated with epcoritamab were identified from the FAERS database. The flow diagram illustrating data collection and analysis of epcoritamab-associated AEs from the FAERS database is presented in Figure 1. The number and signal strength of epcoritamab at the SOC level from the FAERS database are described in Supplementary Table S5. A total of 46 signals at the PT level were detected after conforming to the four algorithms simultaneously. Among these, 9 signals related to the SOC of “neoplasms benign, malignant and unspecified (incl cysts and polyps)” and 2 signals related to the SOC of “injury, poisoning and procedural complications” were considered as epcoritamab-unrelated signals. The number and signal strength of 11 epcoritamab-unrelated signals at the PT level from the FAERS database are listed in Supplementary Table S6. After excluding the 11 epcoritamab-unrelated signals, 35 epcoritamab-associated signals were detected, as shown in Supplementary Table S7.

A total of 513 AE reports and 1,073 AEs associated with epcoritamab were identified from the JADER database. The number and signal strength of epcoritamab at the SOC level from the JADER database are described in Supplementary Table S8. Only 9 signals at the PT level were detected from the JADER database, and 4 epcoritamab-unrelated signals were excluded, as described in Supplementary Table S9. Number and signal strength of 5 epcoritamab-related signals at the PT level from the JADER database are described in Supplementary Table S10. From both the FAERS and JADER databases, CRS and ICANS emerged as the most common and significant epcoritamab-associated AEs. Significant AEs related to the SOC of infections and infestations were also identified.

### 3.2 Subgroup analysis

**Figure 2:**
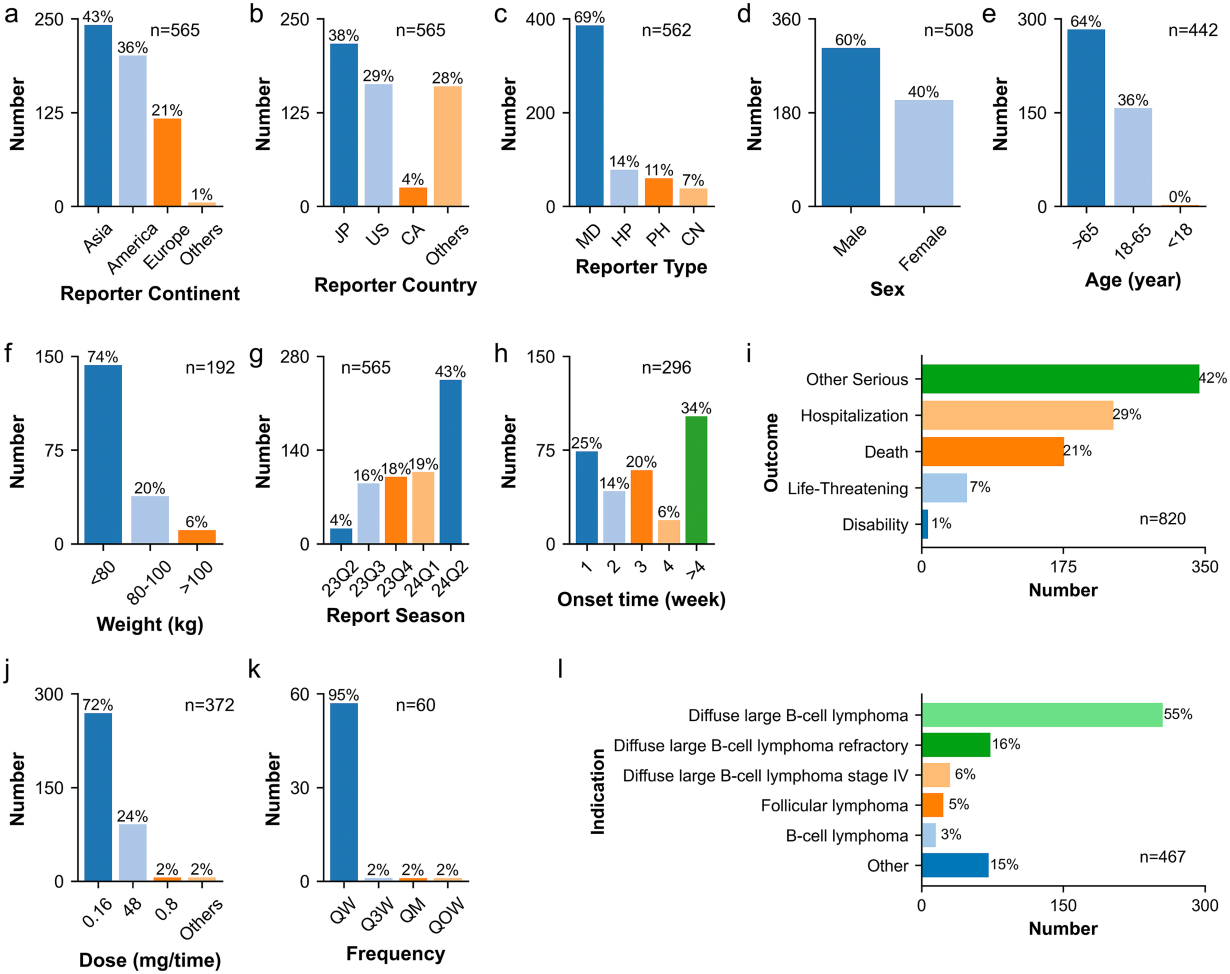
Clinical characteristics of epcoritamab-associated adverse event reports from the FAERS database. **(a)** Reporter continent. **(b)** Reporter country. **(c)** Reporter type. **(d)** Sex. **(e)** Age. **(f)** Weight. **(g)** Report season. **(h)** Onset time. **(i)** Outcome. **(j)** Dose. **(k)** Frequency. **(l)** Indication. JP, Japan; US, United States; CA, Canada; MD, Physician; HP, Health-professional; PH, pharmacist; CN, consumer; QW, once a week; Q3W, once every 3 weeks; QM, once a month; QOW, every other week.

The clinical characteristics of all epcoritamab-associated AE reports from the FAERS database are illustrated in Figure 2. The clinical characteristics of epcoritamab-associated CRS and ICANS reports from the FAERS database are illustrated in Supplementary Figures S1, S2. A total of 4 epcoritamab-associated AEs met the threshold with at least 20 cases in the overall reported number from the FAERS database. The significance level of Bonferroni correction was adjusted to 0.05/4 = 0.0125.

**Figure 3:**
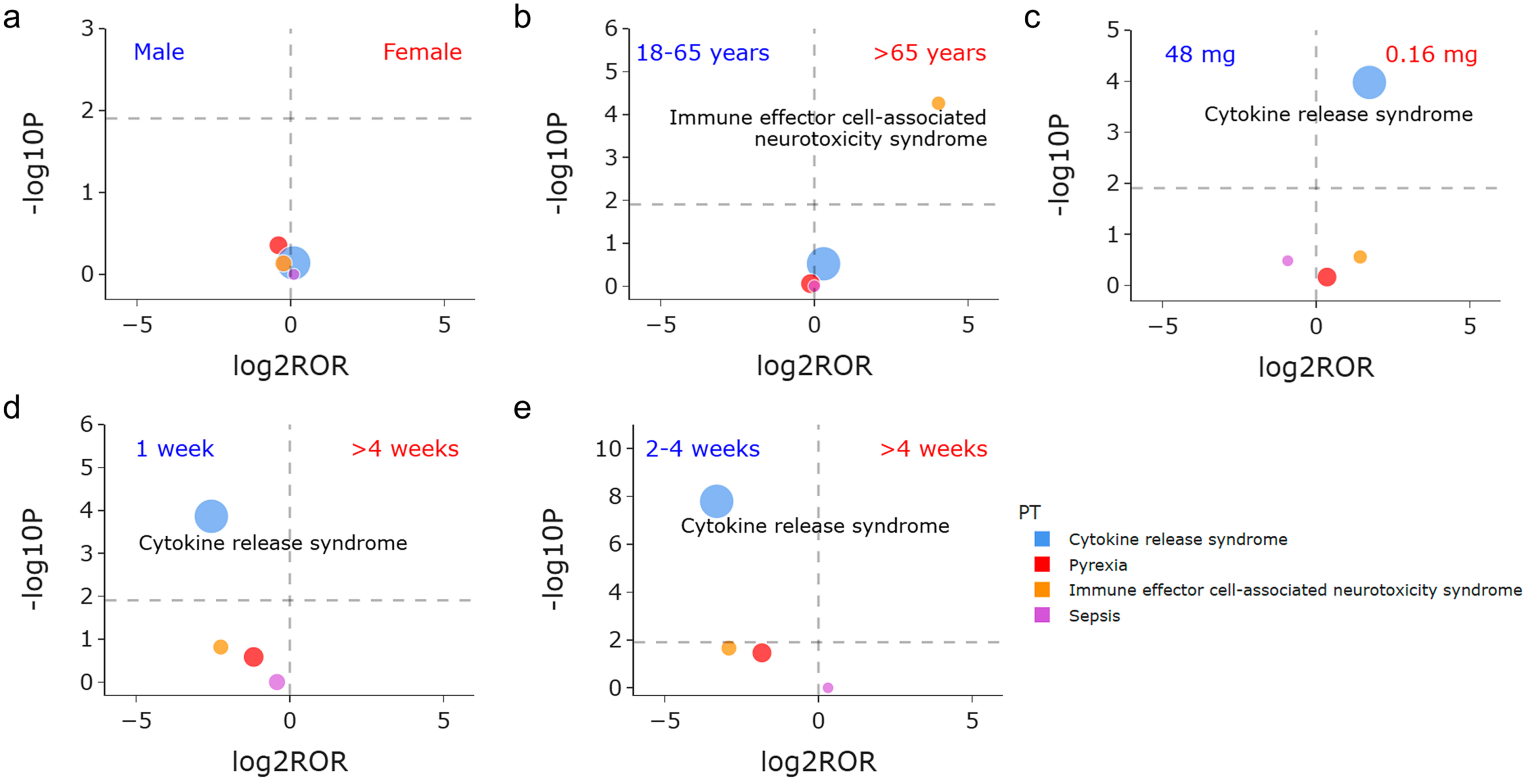
Volcano plots for subgroup analysis of epcoritamab-associated AEs. **(a)** Subgroups of female and male patients. **(b)** Subgroups of patients with age >65 years and 18–65 years. **(c)** Subgroups of patients taking signal dose 0.16 mg and 48 mg. **(d)** Subgroups of onset time >4 weeks and <=1 week. **(e)** Subgroups of onset time >4 weeks and 2–4 weeks. The sizes of each point represent the number of reports of each PT induced by epcoritamab.

Based on the clinical characteristics of the epcoritamab-associated AE reports, the potential differences in AEs across sex, age, dose, and onset time were specifically investigated due to the potential bias from different countries and insufficient subgroup data on other subgroups to support stable inference. The volcano plots in Figure 3 illustrate the disparities in epcoritamab-associated AEs across subgroups. There was no significant difference across female and male patients, as shown in Figure 3a. Figure 3b demonstrated that ICANS was reported with a significantly higher frequency among patients over the age of 65 compared to those aged 18–65 (>65 years vs. 18–65 years: ICANS, ROR 16.439, 95% CI 2.220–121.728; *p* < 0.001).

Concerning CRS signal, a dose of 0.16 mg was significantly more associated with reporting CRS (0.16 mg vs. 48 mg: CRS, ROR 3.323, 95% CI 1.705–6.477, *p* < 0.001), as shown in Figure 3c. Compared with onset time over 4 weeks of medication, CRS was more frequently to manifest within the first week and 2–4 weeks (>4 weeks vs. <=1 week: CRS, ROR 0.171, 95% CI 0.059–0.491, *p* < 0.001; >4 weeks vs. 2–4 weeks: CRS, ROR 0.101, 95% CI 0.036–0.283, *p* < 0.001), as revealed by Figures 3d,e.

### 3.3 Sensitivity analysis

The results of sensitivity analysis, which were stratified by patients over 65 years and 18–65 years, are shown in Supplementary Tables S11, S12. For patients over 65 years, there were significant signal of ICANS, but for patients aged 18–65 years, it became not significant [Over 65 years vs. 18–65 years: ICANS, 91.02 (58.30–142.11) vs. not significant]. These findings are consistent with the results of the subgroup analysis, highlighting the potential importance of monitoring ICANS to older patients.

The results of sensitivity analysis, which were restricted to patients taking 0.16 mg and 48 mg dose of epcoritamab, are shown in Supplementary Tables S13, S14. The patients taking 0.16 mg of epcoritamab had higher signal strength of CRS than those of 48 mg [0.16 mg vs. 48 mg: CRS, 219.77 (178.04–271.29) vs. 64.71 (34.31–122.07)]. The results of sensitivity analysis, which were restricted to reports occurred within the first week, 2–4 weeks, and after 4 weeks of epcoritamab, are shown in Supplementary Tables S15–S17. Within the first week and 2–4 weeks of medication, there were higher signal strength of CRS than those after 4 weeks [1 week vs. 2–4 weeks vs. after 4 weeks: CRS, 179.95 (124.41–260.30) vs. 306.09 (228.36–410.28) vs. 30.48 (11.32–82.13)]. These findings are consistent with the results of the subgroup analysis, indicating heightened attention for CRS at a dose of 0.16 mg and within 4 weeks of medication.

## 4 Discussion

Since the approval of epcoritamab, reports of AEs have continued to increase over the quarters, highlighting the vital for ongoing epidemiological surveillance. Moreover, most of the reports led to serious outcomes, such as hospitalization, death, life-threatening and disability. A thorough analysis of serious AEs, particularly those with potential heterogeneity across subgroups, is essential to identify areas of concern that merit further investigation.

In this study, the FAERS database identified 35 epcoritamab-associated signals, whereas the JADER database yielded only 5. FAERS represents the world’s largest spontaneous reporting system for drug-related AEs (36), while JADER is a post-marketing surveillance database maintained by the Pharmaceutical and Medical Device General Organization (37). Unlike FAERS, which captures reports globally, JADER predominantly receives submissions from Asian countries (38). Although this study used the data in the JADER database spanned the same period with those in the FAERS database to reduce the deviations in different periods, there were still substantial differences in regulatory context and geographic coverage between the two databases. Therefore, this study deliberately refrained from making direct quantitative comparisons of potential heterogeneity across subgroup between the two databases. Instead, the JADER database was treated as a supplementary analysis, intended to provide ancillary contextual information. This study then performed clinical characteristics analysis, subgroup analysis and sensitivity analysis on epcoritamab-associated AEs based on the FAERS database.

The primary objective of this study was to identify potential differences in AEs that warrant further investigation. It must be emphasized that due to the inherent limitations of the FAERS and JADER database, the observed differences in AE reports related to epcoritamab may be influenced by confounding factors, such as reporting bias, data missingness, indications, and polypharmacy. Therefore, these findings should be regarded as preliminary results and require further verification.

### 4.1 CRS

As revealed by the results of signal detection and sensitivity analysis, CRS was the one of the most common and significant epcoritamab-associated AEs, which was detected in all subgroups. The results were consistent with the product label and clinical trials (21, 39, 40). Because of it serious or life-threatening reactions, the United States prescribing information for epcoritamab includes a black box warning regarding the risk of CRS (40), highlighting the requirement for careful monitoring. However, potential differences in CRS across demographic and dosing regimen subgroups are not yet fully understood.

In this study, CRS was more frequently observed within 4 weeks and at a dose of 0.16 mg. This aligns with the known “first-dose effect” seen with many CRS-inducing agents, where the most severe symptoms occur following the initial administration (41). However, it is still a fundamental challenge in pharmacovigilance analysis of step-up dosing regimens: dose and treatment timing are inherently confounded, because the 0.16 mg priming dose is invariably administered at the initial phase of administration. Typically, the 0.16 mg priming dose is invariably administered at the first week, higher doses (0.8 mg and 3 mg) are given in the second week and third week, whereas full dose (48 mg) is given in the fourth week and subsequent cycles. To partially separate dose effects from timing effects, this study analyzed the clinical characteristics of CRS reports. This analysis revealed that 35% of CRS reports occurred within the first week, 25% occurred within the second week, 32% occurred within the third week. Then, this study performed a sensitivity analysis to further verify the potential differences. Specifically, the patients taking 0.16 mg of epcoritamab had higher signal strength of CRS than those of 48 mg. Within the first week and 2–4 weeks of medication, there were higher signal strength of CRS than those after 4 weeks. The clinical characteristics, subgroup analysis and sensitivity analysis consistently indicated that there was still a high frequency of CRS reports at 2–4 weeks. In clinical, the step-up dosing regimen utilized by epcoritamab were designed to mitigate the risk of CRS (42). The priming dose (0.16 mg) was specifically designed to mitigate CRS by inducing gradual T-cell engagement and cytokine release, rather than causing maximal cytokine release. The results suggest that the 0.16 mg priming dose itself may be not the primary driver of CRS; rather, it is the first exposure to T-cell engagement-regardless of the specific dose-that triggers the cytokine cascade. In other words, the observed CRS signal likely reflects the treatment timing effect (first T-cell engagement) rather than a dose-specific effect of the 0.16 mg concentration. Although the findings support a first-exposure interpretation, this study cannot exclude the possibility that the 0.16 mg concentration itself contributes independently to CRS risk. Moreover, due to insufficient number of reports at 0.8 mg and other doses, only the signal of CRS at 0.16 and 48 mg doses were analyzed. This distinction would require larger sample sizes and longer-term studies, or through prospectively designed studies with dose-exposure modeling. Based on the current evidence, this study at least suggest that enhanced CRS monitoring should be prioritized during the initial administration of epcoritamab treatment.

### 4.2 ICANS

ICANS was recognized as another significant and frequently reported epcoritamab-associated AEs from both the FAERS and JADER databases. Neurotoxicity is a potentially life-threatening complication associated with BiTEs therapy and was prominently highlighted in the black box warning of the United States prescribing information (43). Neurotoxicity often requires prolonged hospitalization and supportive care; identifying its key predictors enables early preventive management and targeted intervention for high-risk patients (44). In this study, ICANS exhibited a significant signal among patients over the age of 65, but for patients 18–65 years, it became not significant. Advanced age, existence and severity of CRS, elevated inflammatory markers (C-reactive protein, ferritin), low baseline Montreal Cognitive Assessment (MoCA) score, and impaired baseline attention testing are established risk factors for ICANS (45, 46). Developing models for early ICANS identification and risk prediction is a key research priority moving forward (45). This study recommends close monitoring of AEs and cognitive assessment, with special emphasis on patients aged over 65 years. The finding suggests further investigations to confirm the potential influence of age on the occurrence of ICANS.

### 4.3 Infections and infestations

Except for CRS and ICANS, significant AEs related to the SOC of infections and infestations were also identified from both the FAERS and JADER databases. Sepsis, an infection-related AEs often associated with fatal outcomes, demands that healthcare professionals maintain a high level of awareness regarding its early signs, understand its severe implications, and initiate timely therapeutic interventions (47). The increased susceptibility to infections among patients undergoing BiTE therapy may be attributed to factors such as immunosuppression, neutropenia, and T cell exhaustion (48). The management of CRS, particularly severe cases (grade ≥3), often involves immunosuppressive agents like corticosteroids, interleukin-6 inhibitors, and tumor necrosis factor alpha inhibitors, which may exacerbate immunosuppression and further increase infection risk (49, 50). Due to the small number of reports, the potential heterogeneity of epcoritamab-associated infection and infestation events in demographics and dosing regimens were not detected in this study.

### 4.4 Limitations

While this study benefits from the strengths of large-scale real-world research and data mining techniques, certain limitations should be consideration. Firstly, the FAERS and JADER operate as spontaneous reporting systems, which may yield missing data and introduce bias. Across key variables, the proportion of incomplete reporting may be non-negligible. Missingness is unlikely to be completely at random, as it may reflect reporter characteristics, event severity, and regional reporting practices. For stance, weight may be more frequently missing in reports where the AE is less severe or where the reporter is a non-physician, which could bias weight-related subgroup analysis toward more complete reporting in serious events. Additionally, due to the small number of reports on weight, the potential differences in AEs across weight subgroups were not investigated. For other subgroups, while this study produced sensitivity analysis, the possibility that selective reporting or differential missingness has influenced the subgroup-specific estimates still cannot be excluded. Secondly, the study is unable to calculate the incidence rate of AEs due to the absence of comprehensive data regarding the total number of patients treated with epcoritamab. Thirdly, it is important to note the possibility of false positives arising from multiple testing increased (51). To reduce the risk of false positives, this study involved multiple comparisons with Bonferroni correction. However, Bonferroni correction may obscure clinically meaningful but weaker signals (51). Fourthly, CRS events could be attributed either to the specific dose level or to the treatment timing, and these two effects cannot be fully disentangled using spontaneous reporting data alone. Fifthly, the result may be affected by the drugs being used simultaneously. For instance, certain traditional Chinese medicines may have anti-tumor activity or nervous system and immune regulation effects (52–57). The combined use of these drugs may influence the AEs associated with epcoritamab. However, this study did not analyze the impact of this confounding factor. Sixthly, disproportionality analysis does not quantify risk or establish causality between the drug and AEs (58, 59). Therefore, further experimental investigations are necessary to corroborate these findings. Notwithstanding these limitations, the results of this study may serve as a valuable resource for healthcare professionals in monitoring patients and tracking the AEs associated with epcoritamab.

## 5 Conclusion

This study revealed the potential heterogeneity of epcoritamab associated AEs in demographics and dosing regimens. Enhanced CRS monitoring should be prioritized during the initial administration of epcoritamab treatment. Monitoring ICANS may be of particular importance in older patients. Further research is warranted to elucidate the case causality of these associations.

## Data Availability

The original contributions presented in the study are included in the article/Supplementary material, further inquiries can be directed to the corresponding authors.
