# Supplementary material for: Potential heterogeneity of epcoritamab associated adverse events in demographics and dosing regimens
Source: Front Med (Lausanne). 2026 Jul 23;13:1832550. doi: 10.3389/fmed.2026.1832550 (PMC13441769; doi:10.3389/fmed.2026.1832550)
Supplement: Supplementary file 1 [file Table_1.docx]

# Supplementary Material

## Supplementary Table S1. Fourfold table of disproportionality analysis for epcoritamab signal detection.

|  | Target adverse event | Non-target adverse events | Total |
| --- | --- | --- | --- |
| Epcoritamab | a | b | a+b |
| All other drugs | c | d | c+d |
| Total | a+c | b+d | a+b+c+d |

For specific details, please refer to the references cited in the Method section of the manuscript.

## Supplementary Table S2. Equations and criteria of four algorithms for epcoritamab signal detection.

| Algorithms | Equation | Criteria |
| --- | --- | --- |
| ROR | ROR=(ad)/(bc) | Lower limit of 95% CI>1, N≥2 |
|  | Lower limit of 95% CI=e^ln(ROR)-1.96(1/a+1/b+1/c+1/d)^0.5^  Upper limit of 95% CI=e^ln(ROR)+1.96(1/a+1/b+1/c+1/d)^0.5^ |  |
| PRR | PRR=a(c+d)/c/(a+b) | PRR≥2, χ2≥4, N≥3 |
|  | χ2=[(ad-bc)^2](a+b+c+d)/[(a+b)(c+d)(a+c)(b+d)] |  |
| BCPNN | IC=log_2_a(a+b+c+d)/(a+c)/(a+b) | IC025>0 |
|  | IC025=E(IC)-2V(IC)^0.5 |  |
| MGPS | EBGM=a(a+b+c+d)/(a+c)/(a+b) | EBGM05>2 |
|  | EBGM05=e^ln(EBGM)-1.96(1/a+1/b+1/c+1/d)^0.5^ |  |

**Abbreviations:** ROR, reporting odds ratio; CI, confidence interval; N, number of co-occurrences; PRR, proportional reporting ratio; χ2, chi-squared; BCPNN, Bayesian confidence propagation neural network; IC, information component; IC025, lower limit of 95% confidence interval of IC; E(IC), IC expectation; V(IC), variance of IC; MGPS, multi-item gamma Poisson shrinker; EBGM, empirical Bayesian geometric mean; EBGM05, lower limit of 95% confidence interval of EBGM. For specific details, please refer to the references cited in the Method section of the manuscript.

## Supplementary Table S3. Fourfold table of subgroup analysis of epcoritamab signals.

|  | Target adverse event with epcoritamab | Non-target adverse events with epcoritamab | Total |
| --- | --- | --- | --- |
| Subgroup 1 | a | b | a+b |
| Subgroup 2 | c | d | c+d |
| Total | a+c | b+d | a+b+c+d |

For specific details, please refer to the references cited in the Method section of the manuscript.

## Supplementary Table S4. Criteria of ROR and Fisher’s exact test for subgroup analysis of epcoritamab signals.

|  | ROR | Fisher’s exact test |
| --- | --- | --- |
| Signals that subgroup 1 more frequently to report | ROR>1, Lower limit of 95% CI>1, N≥2 | P<0.05 |
| Signals that subgroup 2 more frequently to report | ROR<1, Upper limit of 95% CI<1, N≥2 | P<0.05 |

**Abbreviations:** ROR, reporting odds ratio; 95% CI, 95% confidence interval; N, number of co-occurrences. For specific details, please refer to the references cited in the Method section of the manuscript.

## Supplementary Table S5. Number and signal strength of epcoritamab at the SOC level from the FAERS database.

| SOC | Number | ROR (95% CI) | PRR (χ2) | IC (IC025) | EBGM (EBGM05) |
| --- | --- | --- | --- | --- | --- |
| Neoplasms benign, malignant and unspecified (incl cysts and polyps) (SOC: 10029104) ^abcd^ | 217 | 9.43 (8.17-10.90) | 8.18 (1390.84) | 3.03 (2.77) | 8.17 (7.07) |
| Infections and infestations (SOC: 10021881) ^abcd^ | 210 | 2.55 (2.20-2.95) | 2.32 (168.66) | 1.22 (0.99) | 2.32 (2.01) |
| General disorders and administration site conditions (SOC: 10018065) | 184 | 0.67 (0.57-0.78) | 0.71 (26.09) | -0.49 (-0.71) | 0.71 (0.61) |
| Immune system disorders (SOC: 10021428) ^abcd^ | 172 | 10.98 (9.36-12.87) | 9.81 (1373.30) | 3.29 (2.99) | 9.78 (8.34) |
| Investigations (SOC: 10022891) ^ac^ | 119 | 1.40 (1.16-1.68) | 1.36 (12.23) | 0.45 (0.17) | 1.36 (1.13) |
| Nervous system disorders (SOC: 10029205) | 97 | 0.92 (0.75-1.13) | 0.92 (0.68) | -0.12 (-0.42) | 0.92 (0.75) |
| Gastrointestinal disorders (SOC: 10017947) | 62 | 0.51 (0.39-0.65) | 0.53 (28.74) | -0.93 (-1.29) | 0.53 (0.41) |
| Respiratory, thoracic and mediastinal disorders (SOC: 10038738) | 58 | 0.86 (0.66-1.11) | 0.86 (1.32) | -0.21 (-0.59) | 0.86 (0.66) |
| Injury, poisoning and procedural complications (SOC: 10022117) | 56 | 0.25 (0.19-0.32) | 0.28 (122.70) | -1.85 (-2.22) | 0.28 (0.21) |
| Blood and lymphatic system disorders (SOC: 10005329) ^ac^ | 44 | 1.78 (1.32-2.40) | 1.75 (14.50) | 0.81 (0.35) | 1.75 (1.30) |
| Vascular disorders (SOC: 10047065) | 36 | 1.37 (0.98-1.91) | 1.36 (3.50) | 0.44 (-0.05) | 1.36 (0.98) |
| Musculoskeletal and connective tissue disorders (SOC: 10028395) | 32 | 0.40 (0.29-0.57) | 0.42 (27.41) | -1.26 (-1.74) | 0.42 (0.29) |
| Cardiac disorders (SOC: 10007541) | 31 | 1.14 (0.80-1.63) | 1.14 (0.53) | 0.19 (-0.34) | 1.14 (0.80) |
| Psychiatric disorders (SOC: 10037175) | 27 | 0.35 (0.24-0.52) | 0.37 (31.39) | -1.45 (-1.97) | 0.37 (0.25) |
| Metabolism and nutrition disorders (SOC: 10027433) | 25 | 0.87 (0.58-1.29) | 0.87 (0.49) | -0.20 (-0.76) | 0.87 (0.59) |
| Surgical and medical procedures (SOC: 10042613) | 24 | 0.97 (0.65-1.45) | 0.97 (0.02) | -0.04 (-0.62) | 0.97 (0.65) |
| Renal and urinary disorders (SOC: 10038359) | 22 | 0.95 (0.62-1.45) | 0.95 (0.05) | -0.07 (-0.67) | 0.95 (0.63) |
| Hepatobiliary disorders (SOC: 10019805) | 16 | 1.25 (0.76-2.04) | 1.24 (0.76) | 0.31 (-0.41) | 1.24 (0.76) |
| Skin and subcutaneous tissue disorders (SOC: 10040785) | 15 | 0.18 (0.11-0.30) | 0.19 (54.00) | -2.38 (-3.03) | 0.19 (0.12) |
| Eye disorders (SOC: 10015919) | 9 | 0.30 (0.15-0.57) | 0.30 (14.76) | -1.72 (-2.54) | 0.30 (0.16) |
| Endocrine disorders (SOC: 10014698) | 3 | 0.66 (0.21-2.06) | 0.66 (0.51) | -0.59 (-1.91) | 0.66 (0.21) |
| Social circumstances (SOC: 10041244) | 2 | 0.28 (0.07-1.11) | 0.28 (3.76) | -1.84 (-3.12) | 0.28 (0.07) |
| Reproductive system and breast disorders (SOC: 10038604) | 1 | 0.12 (0.02-0.85) | 0.12 (6.51) | -3.06 (-4.27) | 0.12 (0.02) |
| Congenital, familial and genetic disorders (SOC: 10010331) | 1 | 0.32 (0.05-2.28) | 0.32 (1.44) | -1.64 (-3.08) | 0.32 (0.05) |
| Ear and labyrinth disorders (SOC: 10013993) | 1 | 0.16 (0.02-1.11) | 0.16 (4.54) | -2.67 (-3.92) | 0.16 (0.02) |

^a^: SOCs met the criteria of ROR algorithm; ^b^: SOCs met the criteria of PRR algorithm; ^c^: SOCs met the criteria of BCPNN algorithm; ^d^: SOCs met the criteria of MGPS algorithm. **Abbreviations:** SOC, system organ class; ROR, reporting odds ratio; CI, confidence interval; PRR, proportional reporting ratio; χ2, chi-squared; IC, information component; IC025, lower limit of 95% confidence interval of IC; EBGM, empirical Bayesian geometric mean; EBGM05, lower limit of 95% confidence interval of EBGM.

## Supplementary Table S6. Number and signal strength of 11 epcoritamab-unrelated signals at the PT level from the FAERS database.

| PT | Number | ROR (95% CI) | PRR (χ2) | IC (IC025) | EBGM (EBGM05) |
| --- | --- | --- | --- | --- | --- |
| Neoplasms benign, malignant and unspecified (incl cysts and polyps) (SOC: 10029104) | | | | | |
| Diffuse large B-cell lymphoma (PT: 10012818) | 119 | 535.43 (439.47-652.35) | 491.99 (52079.03) | 8.78 (6.27) | 439.45 (360.69) |
| Diffuse large B-cell lymphoma refractory (PT: 10012822) | 36 | 1352.85 (926.01-1976.43) | 1319.61 (35896.83) | 9.96 (4.62) | 998.87 (683.71) |
| Malignant neoplasm progression (PT: 10051398) | 16 | 6.42 (3.92-10.52) | 6.37 (72.38) | 2.67 (1.57) | 6.36 (3.88) |
| Tumour associated fever (PT: 10052243) | 5 | 457.72 (181.43-1154.75) | 456.16 (2043.75) | 8.68 (1.32) | 410.64 (162.77) |
| B-cell lymphoma (PT: 10003899) | 4 | 73.51 (27.31-197.84) | 73.31 (280.31) | 6.17 (0.94) | 72.04 (26.77) |
| Lymphoma (PT: 10025310) | 3 | 9.72 (3.13-30.20) | 9.70 (23.35) | 3.27 (0.16) | 9.68 (3.11) |
| High-grade B-cell lymphoma (PT: 10080215) | 3 | 373.99 (114.57-1220.79) | 373.22 (1020.88) | 8.42 (0.47) | 342.20 (104.83) |
| Primary mediastinal large B-cell lymphoma (PT: 10036710) | 3 | 881.54 (253.07-3070.81) | 879.74 (2168.54) | 9.50 (0.40) | 724.67 (208.03) |
| Tumour pain (PT: 10045171) | 3 | 86.30 (27.48-271.08) | 86.13 (247.24) | 6.40 (0.49) | 84.38 (26.86) |
| Injury, poisoning and procedural complications (SOC: 10022117) | | | | | |
| Intentional underdose (PT: 10073954) | 5 | 63.37 (26.16-153.51) | 63.16 (301.26) | 5.96 (1.28) | 62.22 (25.69) |
| Intercepted product preparation error (PT: 10079213) | 4 | 5.44 (2.04-14.52) | 5.43 (14.43) | 2.44 (0.23) | 5.42 (2.03) |

**Abbreviations:** PT, preferred term; ROR, reporting odds ratio; CI, confidence interval; PRR, proportional reporting ratio; χ2, chi-squared; IC, information component; IC025, lower limit of 95% confidence interval of IC; EBGM, empirical Bayesian geometric mean; EBGM05, lower limit of 95% confidence interval of EBGM.

## Supplementary Table S7. Number and signal strength of 35 epcoritamab-associated signals at the PT level from the FAERS database.

| PT | Number | ROR (95% CI) | PRR (χ2) | IC (IC025) | EBGM (EBGM05) |
| --- | --- | --- | --- | --- | --- |
| Immune system disorders (SOC: 10021428) | | | | | |
| Cytokine release syndrome (PT: 10052015) | 159 | 177.23 (149.91-209.54) | 158.09 (23916.54) | 7.25 (6.05) | 152.27 (128.79) |
| Haemophagocytic lymphohistiocytosis (PT: 10071583) | 7 | 20.61 (9.79-43.38) | 20.51 (129.31) | 4.35 (1.55) | 20.42 (9.70) |
| Infections and infestations (SOC: 10021881) | | | | | |
| Sepsis (PT: 10040047) | 20 | 8.83 (5.67-13.73) | 8.72 (136.58) | 3.12 (2.03) | 8.70 (5.59) |
| Infection (PT: 10021789) | 16 | 4.14 (2.53-6.78) | 4.10 (37.63) | 2.04 (1.09) | 4.10 (2.51) |
| Cytomegalovirus infection (PT: 10011831) | 15 | 42.00 (25.19-70.03) | 41.58 (588.27) | 5.36 (2.82) | 41.17 (24.69) |
| COVID-19 pneumonia (PT: 10084380) | 8 | 14.35 (7.16-28.79) | 14.28 (98.49) | 3.83 (1.56) | 14.23 (7.10) |
| Bacteraemia (PT: 10003997) | 7 | 24.32 (11.55-51.22) | 24.21 (154.88) | 4.59 (1.60) | 24.07 (11.43) |
| Cytomegalovirus viraemia (PT: 10058854) | 6 | 43.39 (19.38-97.15) | 43.22 (244.88) | 5.42 (1.52) | 42.78 (19.11) |
| Septic shock (PT: 10040070) | 6 | 6.12 (2.75-13.66) | 6.10 (25.58) | 2.61 (0.72) | 6.10 (2.73) |
| Cytomegalovirus infection reactivation (PT: 10058666) | 5 | 21.34 (8.85-51.47) | 21.27 (96.11) | 4.40 (1.09) | 21.17 (8.78) |
| Pneumonia cytomegaloviral (PT: 10035676) | 5 | 111.33 (45.73-271.06) | 110.96 (530.51) | 6.76 (1.32) | 108.06 (44.39) |
| Pneumonia bacterial (PT: 10060946) | 4 | 13.01 (4.87-34.78) | 12.98 (44.10) | 3.69 (0.64) | 12.94 (4.84) |
| Pneumocystis jirovecii pneumonia (PT: 10073755) | 3 | 10.10 (3.25-31.39) | 10.08 (24.48) | 3.33 (0.18) | 10.06 (3.24) |
| Bronchopulmonary aspergillosis (PT: 10006473) | 3 | 15.09 (4.85-46.93) | 15.06 (39.23) | 3.91 (0.29) | 15.01 (4.82) |
| Escherichia infection (PT: 10061126) | 3 | 16.15 (5.19-50.25) | 16.12 (42.39) | 4.01 (0.30) | 16.06 (5.16) |
| Pneumonia viral (PT: 10035737) | 3 | 34.38 (11.02-107.21) | 34.31 (96.21) | 5.09 (0.43) | 34.03 (10.91) |
| Progressive multifocal leukoencephalopathy (PT: 10036807) | 3 | 16.56 (5.32-51.54) | 16.53 (43.61) | 4.04 (0.31) | 16.47 (5.29) |
| General disorders and administration site conditions (SOC: 10018065) | | | | | |
| Pyrexia (PT: 10037660) | 48 | 6.26 (4.69-8.35) | 6.09 (204.86) | 2.60 (2.04) | 6.08 (4.56) |
| Investigations (SOC: 10022891) | | | | | |
| Neutrophil count decreased (PT: 10029366) | 16 | 13.24 (8.08-21.69) | 13.11 (178.50) | 3.71 (2.23) | 13.07 (7.98) |
| Platelet count decreased (PT: 10035528) | 13 | 4.81 (2.79-8.31) | 4.78 (38.87) | 2.26 (1.13) | 4.77 (2.76) |
| C-reactive protein increased (PT: 10006825) | 9 | 8.48 (4.40-16.35) | 8.44 (58.91) | 3.07 (1.36) | 8.42 (4.37) |
| Blood lactate dehydrogenase increased (PT: 10005630) | 7 | 25.57 (12.14-53.86) | 25.45 (163.47) | 4.66 (1.62) | 25.30 (12.01) |
| Cytomegalovirus test positive (PT: 10051620) | 3 | 88.79 (28.26-278.98) | 88.61 (254.37) | 6.44 (0.49) | 86.76 (27.61) |
| Nervous system disorders (SOC: 10029205) | | | | | |
| Immune effector cell-associated neurotoxicity syndrome (PT: 10083347) | 37 | 107.60 (77.32-149.73) | 104.90 (3713.86) | 6.68 (4.32) | 102.32 (73.53) |
| Neurotoxicity (PT: 10029350) | 5 | 11.66 (4.84-28.09) | 11.62 (48.43) | 3.54 (0.88) | 11.59 (4.81) |
| Vascular disorders (SOC: 10047065) | | | | | |
| Hypotension (PT: 10021097) | 19 | 4.75 (3.02-7.47) | 4.70 (55.49) | 2.23 (1.34) | 4.70 (2.99) |
| Respiratory, thoracic and mediastinal disorders (SOC: 10038738) | | | | | |
| Pleural effusion (PT: 10035598) | 8 | 6.80 (3.39-13.63) | 6.77 (39.29) | 2.76 (1.08) | 6.76 (3.37) |
| Hypoxia (PT: 10021143) | 6 | 7.86 (3.52-17.54) | 7.83 (35.71) | 2.97 (0.89) | 7.82 (3.50) |
| Blood and lymphatic system disorders (SOC: 10005329) | | | | | |
| Febrile neutropenia (PT: 10016288) | 10 | 5.82 (3.12-10.84) | 5.78 (39.56) | 2.53 (1.14) | 5.78 (3.10) |
| Disseminated intravascular coagulation (PT: 10013442) | 3 | 14.47 (4.65-45.00) | 14.44 (37.40) | 3.85 (0.28) | 14.39 (4.63) |
| Cardiac disorders (SOC: 10007541) | | | | | |
| Atrial fibrillation (PT: 10003658) | 10 | 5.04 (2.70-9.39) | 5.01 (32.12) | 2.32 (1.00) | 5.01 (2.69) |
| Gastrointestinal disorders (SOC: 10017947) | | | | | |
| Gastrointestinal haemorrhage (PT: 10017955) | 5 | 5.44 (2.26-13.10) | 5.42 (18.03) | 2.44 (0.46) | 5.42 (2.25) |
| Ascites (PT: 10003445) | 4 | 6.95 (2.60-18.55) | 6.93 (20.28) | 2.79 (0.37) | 6.92 (2.59) |
| Metabolism and nutrition disorders (SOC: 10027433) | | | | | |
| Tumour lysis syndrome (PT: 10045170) | 5 | 20.21 (8.38-48.73) | 20.14 (90.54) | 4.33 (1.08) | 20.05 (8.32) |
| Psychiatric disorders (SOC: 10037175) | | | | | |
| Delirium (PT: 10012218) | 5 | 6.20 (2.58-14.93) | 6.18 (21.70) | 2.63 (0.55) | 6.18 (2.56) |

**Abbreviations:** PT, preferred term; ROR, reporting odds ratio; CI, confidence interval; PRR, proportional reporting ratio; χ2, chi-squared; IC, information component; IC025, lower limit of 95% confidence interval of IC; EBGM, empirical Bayesian geometric mean; EBGM05, lower limit of 95% confidence interval of EBGM.

## Supplementary Table S8. Number and signal strength of epcoritamab at the SOC level from the JADER database.

| SOC | Number | ROR (95% CI) | PRR (χ2) | IC (IC025) | EBGM (EBGM05) |
| --- | --- | --- | --- | --- | --- |
| Immune system disorders (SOC: 10021428) ^abcd^ | 546 | 36.80 (32.55-41.60) | 18.57 (8516.20) | 4.09 (3.89) | 16.99 (15.03) |
| Neoplasms benign, malignant and unspecified (incl cysts and polyps) (SOC: 10029104) ^abc^ | 145 | 2.47 (2.07-2.95) | 2.27 (108.81) | 1.18 (0.91) | 2.26 (1.89) |
| Infections and infestations (SOC: 10021881) | 85 | 0.77 (0.62-0.96) | 0.79 (5.31) | -0.34 (-0.66) | 0.79 (0.63) |
| Nervous system disorders (SOC: 10029205) | 67 | 0.82 (0.64-1.05) | 0.83 (2.55) | -0.27 (-0.63) | 0.83 (0.65) |
| Investigations (SOC: 10022891) | 55 | 0.75 (0.57-0.98) | 0.76 (4.53) | -0.40 (-0.79) | 0.76 (0.58) |
| Blood and lymphatic system disorders (SOC: 10005329) | 54 | 0.75 (0.57-0.99) | 0.76 (4.30) | -0.39 (-0.79) | 0.76 (0.58) |
| Respiratory, thoracic and mediastinal disorders (SOC: 10038738) | 23 | 0.29 (0.19-0.43) | 0.30 (40.05) | -1.73 (-2.28) | 0.30 (0.20) |
| Gastrointestinal disorders (SOC: 10017947) | 18 | 0.18 (0.11-0.29) | 0.20 (65.01) | -2.35 (-2.95) | 0.20 (0.12) |
| Cardiac disorders (SOC: 10007541) | 15 | 0.33 (0.20-0.55) | 0.34 (19.82) | -1.54 (-2.21) | 0.34 (0.21) |
| Metabolism and nutrition disorders (SOC: 10027433) | 14 | 0.26 (0.16-0.45) | 0.27 (28.22) | -1.86 (-2.54) | 0.28 (0.16) |
| Psychiatric disorders (SOC: 10037175) | 12 | 0.69 (0.39-1.22) | 0.69 (1.64) | -0.52 (-1.30) | 0.70 (0.39) |
| General disorders and administration site conditions (SOC: 10018065) | 12 | 0.18 (0.10-0.31) | 0.18 (45.93) | -2.43 (-3.14) | 0.19 (0.10) |
| Renal and urinary disorders (SOC: 10038359) | 9 | 0.20 (0.10-0.38) | 0.21 (28.71) | -2.27 (-3.07) | 0.21 (0.11) |
| Hepatobiliary disorders (SOC: 10019805) | 5 | 0.12 (0.05-0.29) | 0.12 (32.62) | -3.02 (-3.98) | 0.12 (0.05) |
| Endocrine disorders (SOC: 10014698) | 4 | 0.08 (0.03-0.21) | 0.08 (42.30) | -3.58 (-4.58) | 0.08 (0.03) |
| Skin and subcutaneous tissue disorders (SOC: 10040785) | 3 | 0.06 (0.02-0.18) | 0.06 (45.47) | -4.03 (-5.09) | 0.06 (0.02) |
| Musculoskeletal and connective tissue disorders (SOC: 10028395) | 2 | 0.08 (0.02-0.32) | 0.08 (21.21) | -3.61 (-4.76) | 0.08 (0.02) |
| Injury, poisoning and procedural complications (SOC: 10022117) | 2 | 0.06 (0.01-0.24) | 0.06 (29.58) | -4.02 (-5.15) | 0.06 (0.02) |
| Ear and labyrinth disorders (SOC: 10013993) | 1 | 0.53 (0.07-3.77) | 0.53 (0.42) | -0.91 (-2.58) | 0.53 (0.07) |

^a^: SOCs met the criteria of ROR algorithm; ^b^: SOCs met the criteria of PRR algorithm; ^c^: SOCs met the criteria of BCPNN algorithm; ^d^: SOCs met the criteria of MGPS algorithm. **Abbreviations:** SOC, system organ class; ROR, reporting odds ratio; CI, confidence interval; PRR, proportional reporting ratio; χ2, chi-squared; IC, information component; IC025, lower limit of 95% confidence interval of IC; EBGM, empirical Bayesian geometric mean; EBGM05, lower limit of 95% confidence interval of EBGM.

## Supplementary Table S9. Number and signal strength of 4 epcoritamab-unrelated signals at the PT level from the JADER database.

| PT | Number | ROR (95% CI) | PRR (χ2) | IC (IC025) | EBGM (EBGM05) |
| --- | --- | --- | --- | --- | --- |
| Neoplasms benign, malignant and unspecified (incl cysts and polyps) (SOC: 10029104) | | | | | |
| Diffuse large B-cell lymphoma (PT: 10012818) | 68 | 55.37 (42.04-72.94) | 51.93 (2667.21) | 5.36 (4.30) | 40.93 (31.08) |
| Diffuse large B-cell lymphoma refractory (PT: 10012822) | 31 | 2006.99 (612.61-6575.15) | 1948.98 (5326.46) | 7.43 (4.04) | 172.88 (52.77) |
| Tumour flare (PT: 10045169) | 10 | 173.07 (73.35-408.38) | 171.46 (887.79) | 6.50 (2.23) | 90.29 (38.26) |
| Tumour pseudoprogression (PT: 10077472) | 8 | 20.54 (9.88-42.70) | 20.39 (133.18) | 4.21 (1.63) | 18.50 (8.90) |

**Abbreviations:** PT, preferred term; ROR, reporting odds ratio; CI, confidence interval; PRR, proportional reporting ratio; χ2, chi-squared; IC, information component; IC025, lower limit of 95% confidence interval of IC; EBGM, empirical Bayesian geometric mean; EBGM05, lower limit of 95% confidence interval of EBGM.

## Supplementary Table S10. Number and signal strength of 5 epcoritamab-associated signals at the PT level from the JADER database.

| PT | Number | ROR (95% CI) | PRR (χ2) | IC (IC025) | EBGM (EBGM05) |
| --- | --- | --- | --- | --- | --- |
| Immune system disorders (SOC: 10021428) | | | | | |
| Cytokine release syndrome (PT: 10052015) | 520 | 184.52 (161.29-211.10) | 95.50 (32528.06) | 5.99 (5.66) | 63.73 (55.71) |
| Hypogammaglobulinaemia (PT: 10020983) | 15 | 21.89 (12.79-37.47) | 21.60 (264.56) | 4.28 (2.41) | 19.48 (11.38) |
| Nervous system disorders (SOC: 10029205) | | | | | |
| Immune effector cell-associated neurotoxicity syndrome (PT: 10083347) | 58 | 162.83 (114.48-231.61) | 154.08 (4857.95) | 6.41 (4.67) | 85.25 (59.93) |
| Infections and infestations (SOC: 10021881) | | | | | |
| Cytomegalovirus viraemia (PT: 10058854) | 5 | 6.48 (2.65-15.85) | 6.46 (22.32) | 2.65 (0.53) | 6.28 (2.57) |
| Cytomegalovirus infection reactivation (PT: 10058666) | 4 | 6.05 (2.23-16.41) | 6.04 (16.29) | 2.56 (0.25) | 5.88 (2.17) |

**Abbreviations:** PT, preferred term; ROR, reporting odds ratio; CI, confidence interval; PRR, proportional reporting ratio; χ2, chi-squared; IC, information component; IC025, lower limit of 95% confidence interval of IC; EBGM, empirical Bayesian geometric mean; EBGM05, lower limit of 95% confidence interval of EBGM.

## **Supplementary Figure S1. Clinical characteristics of epcoritamab-associated cytokine release syndrome reports** **from the FAERS database.**

**(a) Reporter continent. (b) Reporter country. (c) Reporter type. (d) Sex. (e) Age. (f) Weight. (g) Report season. (h) Onset time. (i) Outcome. (j) Dose. (k) Frequency. (l) Indication. Abbreviation: JP, Japan; US, United States; FR, France; MD, Physician; PH, pharmacist; HP, Health-professional; CN, consumer; QW, once a week; QM, once a month.**

## **Supplementary Figure S2. Clinical characteristics of epcoritamab-associated immune effector cell-associated neurotoxicity syndrome reports** **from the FAERS database.**

**(a) Reporter continent. (b) Reporter country. (c) Reporter type. (d) Sex. (e) Age. (f) Weight. (g) Report season. (h) Onset time. (i) Outcome. (j) Dose. (k) Frequency. (l) Indication. Abbreviation: JP, Japan; US, United States; IT, Italy; MD, Physician; HP, Health-professional; PH, pharmacist; QW, once a week.**

## Supplementary Table S11. Number and signal strength of epcoritamab-related signals at the PT level stratified by patients over 65 years from the FAERS database.

| PT | Number | ROR (95% CI) | PRR (χ2) | IC (IC025) | EBGM (EBGM05) |
| --- | --- | --- | --- | --- | --- |
| Immune system disorders (SOC: 10021428) | | | | | |
| Cytokine release syndrome (PT: 10052015) | 87 | 111.00 (86.86-141.83) | 98.34 (6820.34) | 6.32 (5.04) | 80.09 (62.67) |
| Haemophagocytic lymphohistiocytosis (PT: 10071583) | 4 | 23.15 (8.44-63.48) | 23.03 (80.00) | 4.45 (0.74) | 21.90 (7.99) |
| Infections and infestations (SOC: 10021881) | | | | | |
| Sepsis (PT: 10040047) | 11 | 7.16 (3.93-13.05) | 7.07 (56.51) | 2.80 (1.37) | 6.97 (3.83) |
| Cytomegalovirus infection (PT: 10011831) | 9 | 59.70 (29.62-120.32) | 59.00 (450.86) | 5.70 (2.11) | 51.95 (25.78) |
| Infection (PT: 10021789) | 9 | 5.01 (2.59-9.70) | 4.96 (28.21) | 2.30 (0.89) | 4.92 (2.54) |
| COVID-19 pneumonia (PT: 10084380) | 5 | 15.20 (6.21-37.20) | 15.11 (63.66) | 3.87 (0.95) | 14.63 (5.98) |
| Septic shock (PT: 10040070) | 5 | 7.05 (2.90-17.11) | 7.01 (25.36) | 2.79 (0.60) | 6.91 (2.85) |
| Bacteraemia (PT: 10003997) | 4 | 21.41 (7.82-58.60) | 21.31 (73.75) | 4.35 (0.73) | 20.34 (7.43) |
| Pneumonia bacterial (PT: 10060946) | 4 | 16.79 (6.17-45.72) | 16.71 (56.87) | 4.01 (0.67) | 16.12 (5.92) |
| Escherichia infection (PT: 10061126) | 3 | 36.67 (11.25-119.48) | 36.53 (95.49) | 5.08 (0.36) | 33.72 (10.35) |
| Cytomegalovirus viraemia (PT: 10058854) | 3 | 41.40 (12.63-135.71) | 41.24 (107.41) | 5.24 (0.36) | 37.69 (11.50) |
| General disorders and administration site conditions (SOC: 10018065) | | | | | |
| Pyrexia (PT: 10037660) | 26 | 7.44 (5.01-11.04) | 7.22 (137.61) | 2.83 (1.96) | 7.11 (4.80) |
| Nervous system disorders (SOC: 10029205) | | | | | |
| Immune effector cell-associated neurotoxicity syndrome (PT: 10083347) | 24 | 91.02 (58.30-142.11) | 88.16 (1714.41) | 6.19 (3.60) | 73.22 (46.90) |
| Investigations (SOC: 10022891) | | | | | |
| Neutrophil count decreased (PT: 10029366) | 12 | 12.33 (6.91-21.98) | 12.15 (119.50) | 3.57 (1.87) | 11.84 (6.64) |
| C-reactive protein increased (PT: 10006825) | 5 | 8.78 (3.61-21.35) | 8.73 (33.57) | 3.10 (0.72) | 8.58 (3.53) |
| Blood lactate dehydrogenase increased (PT: 10005630) | 3 | 29.84 (9.24-96.41) | 29.73 (77.87) | 4.80 (0.34) | 27.86 (8.62) |
| Blood and lymphatic system disorders (SOC: 10005329) | | | | | |
| Lymphadenopathy (PT: 10025197) | 3 | 8.12 (2.58-25.50) | 8.09 (18.31) | 2.99 (0.07) | 7.96 (2.53) |

For both epcoritamab and all other drugs, only reports of patients over 65 years were included. **Abbreviations:** PT, preferred term; ROR, reporting odds ratio; CI, confidence interval; PRR, proportional reporting ratio; χ2, chi-squared; IC, information component; IC025, lower limit of 95% confidence interval of IC; EBGM, empirical Bayesian geometric mean; EBGM05, lower limit of 95% confidence interval of EBGM.

## Supplementary Table S12. Number and signal strength of epcoritamab-related signals at the PT level stratified by patients 18-65 years from the FAERS database.

| PT | Number | ROR (95% CI) | PRR (χ2) | IC (IC025) | EBGM (EBGM05) |
| --- | --- | --- | --- | --- | --- |
| Immune system disorders (SOC: 10021428) | | | | | |
| Cytokine release syndrome (PT: 10052015) | 46 | 145.13 (106.68-197.43) | 131.34 (5788.82) | 7.00 (4.66) | 127.71 (93.88) |
| General disorders and administration site conditions (SOC: 10018065) | | | | | |
| Pyrexia (PT: 10037660) | 18 | 6.22 (3.88-9.96) | 6.02 (75.74) | 2.59 (1.57) | 6.01 (3.75) |
| Death (PT: 10011906) | 12 | 3.88 (2.19-6.89) | 3.81 (25.05) | 1.93 (0.83) | 3.81 (2.15) |
| Infections and infestations (SOC: 10021881) | | | | | |
| Sepsis (PT: 10040047) | 7 | 10.40 (4.92-21.94) | 10.26 (58.45) | 3.36 (1.22) | 10.24 (4.85) |
| Cytomegalovirus infection (PT: 10011831) | 6 | 41.82 (18.63-93.90) | 41.31 (233.98) | 5.36 (1.51) | 40.95 (18.24) |
| Cytomegalovirus infection reactivation (PT: 10058666) | 4 | 43.41 (16.15-116.67) | 43.06 (162.83) | 5.42 (0.89) | 42.67 (15.87) |
| Pneumonia cytomegaloviral (PT: 10035676) | 3 | 187.06 (58.77-595.41) | 185.90 (530.25) | 7.48 (0.49) | 178.70 (56.14) |
| Vascular disorders (SOC: 10047065) | | | | | |
| Hypotension (PT: 10021097) | 8 | 6.30 (3.13-12.67) | 6.21 (35.02) | 2.63 (1.00) | 6.20 (3.08) |
| Respiratory, thoracic and mediastinal disorders (SOC: 10038738) | | | | | |
| Hypoxia (PT: 10021143) | 3 | 11.32 (3.63-35.28) | 11.26 (27.99) | 3.49 (0.21) | 11.23 (3.60) |
| Pleural effusion (PT: 10035598) | 3 | 8.63 (2.77-26.90) | 8.59 (20.09) | 3.10 (0.12) | 8.57 (2.75) |
| Blood and lymphatic system disorders (SOC: 10005329) | | | | | |
| Febrile neutropenia (PT: 10016288) | 4 | 7.40 (2.76-19.81) | 7.35 (21.92) | 2.87 (0.39) | 7.34 (2.74) |
| Metabolism and nutrition disorders (SOC: 10027433) | | | | | |
| Tumour lysis syndrome (PT: 10045170) | 3 | 42.99 (13.74-134.46) | 42.72 (121.13) | 5.40 (0.44) | 42.34 (13.54) |

For both epcoritamab and all other drugs, only reports of patients 18-65 years were included. **Abbreviations:** PT, preferred term; ROR, reporting odds ratio; CI, confidence interval; PRR, proportional reporting ratio; χ2, chi-squared; IC, information component; IC025, lower limit of 95% confidence interval of IC; EBGM, empirical Bayesian geometric mean; EBGM05, lower limit of 95% confidence interval of EBGM.

## Supplementary Table S13. Number and signal strength of epcoritamab-related signals at the PT level restricted to patients taking 0.16 mg dose of epcoritamab from the FAERS database.

| PT | Number | ROR (95% CI) | PRR (χ2) | IC (IC025) | EBGM (EBGM05) |
| --- | --- | --- | --- | --- | --- |
| Immune system disorders (SOC: 10021428) | | | | | |
| Cytokine release syndrome (PT: 10052015) | 102 | 219.77 (178.04-271.29) | 190.72 (18805.29) | 7.54 (5.75) | 186.21 (150.85) |
| Haemophagocytic lymphohistiocytosis (PT: 10071583) | 4 | 22.41 (8.38-59.95) | 22.30 (81.16) | 4.47 (0.79) | 22.24 (8.31) |
| Infections and infestations (SOC: 10021881) | | | | | |
| Cytomegalovirus infection (PT: 10011831) | 11 | 58.81 (32.36-106.87) | 57.98 (611.56) | 5.85 (2.49) | 57.56 (31.67) |
| Infection (PT: 10021789) | 11 | 5.44 (3.00-9.87) | 5.38 (39.28) | 2.43 (1.14) | 5.37 (2.96) |
| Sepsis (PT: 10040047) | 9 | 7.55 (3.91-14.57) | 7.47 (50.47) | 2.90 (1.26) | 7.46 (3.87) |
| Bacteraemia (PT: 10003997) | 6 | 39.83 (17.80-89.12) | 39.53 (224.24) | 5.30 (1.50) | 39.34 (17.58) |
| COVID-19 pneumonia (PT: 10084380) | 5 | 17.10 (7.09-41.24) | 16.99 (75.12) | 4.08 (1.03) | 16.96 (7.03) |
| Pneumonia cytomegaloviral (PT: 10035676) | 4 | 169.20 (62.68-456.72) | 168.32 (651.32) | 7.36 (0.98) | 164.80 (61.05) |
| Cytomegalovirus viraemia (PT: 10058854) | 4 | 55.02 (20.52-147.46) | 54.73 (209.56) | 5.76 (0.92) | 54.36 (20.28) |
| Bronchopulmonary aspergillosis (PT: 10006473) | 3 | 28.81 (9.25-89.72) | 28.71 (79.94) | 4.84 (0.41) | 28.60 (9.19) |
| Progressive multifocal leukoencephalopathy (PT: 10036807) | 3 | 31.64 (10.16-98.54) | 31.52 (88.30) | 4.97 (0.42) | 31.40 (10.08) |
| Pneumonia viral (PT: 10035737) | 3 | 65.66 (21.03-205.00) | 65.41 (188.70) | 6.02 (0.48) | 64.87 (20.78) |
| Pneumonia bacterial (PT: 10060946) | 3 | 18.62 (5.98-57.93) | 18.55 (49.70) | 4.21 (0.33) | 18.51 (5.95) |
| Escherichia infection (PT: 10061126) | 3 | 30.85 (9.91-96.08) | 30.73 (85.97) | 4.94 (0.41) | 30.62 (9.83) |
| Staphylococcal infection (PT: 10058080) | 3 | 10.53 (3.39-32.76) | 10.50 (25.75) | 3.39 (0.19) | 10.48 (3.37) |
| Investigations (SOC: 10022891) | | | | | |
| Neutrophil count decreased (PT: 10029366) | 12 | 19.01 (10.74-33.64) | 18.72 (201.02) | 4.22 (2.18) | 18.68 (10.56) |
| Platelet count decreased (PT: 10035528) | 9 | 6.37 (3.30-12.29) | 6.31 (40.22) | 2.66 (1.12) | 6.30 (3.26) |
| C-reactive protein increased (PT: 10006825) | 8 | 14.43 (7.19-28.98) | 14.29 (98.79) | 3.83 (1.56) | 14.27 (7.11) |
| Blood lactate dehydrogenase increased (PT: 10005630) | 5 | 34.82 (14.42-84.06) | 34.60 (162.47) | 5.11 (1.20) | 34.45 (14.27) |
| Cytomegalovirus test positive (PT: 10051620) | 3 | 169.58 (53.91-533.41) | 168.93 (490.23) | 7.37 (0.51) | 165.38 (52.58) |
| General disorders and administration site conditions (SOC: 10018065) | | | | | |
| Pyrexia (PT: 10037660) | 30 | 7.50 (5.21-10.81) | 7.25 (162.33) | 2.86 (2.06) | 7.24 (5.03) |
| Multiple organ dysfunction syndrome (PT: 10077361) | 3 | 6.89 (2.22-21.43) | 6.87 (15.04) | 2.78 (0.03) | 6.86 (2.21) |
| Nervous system disorders (SOC: 10029205) | | | | | |
| Immune effector cell-associated neurotoxicity syndrome (PT: 10083347) | 18 | 98.32 (61.43-157.35) | 96.04 (1672.73) | 6.57 (3.32) | 94.88 (59.29) |
| Respiratory, thoracic and mediastinal disorders (SOC: 10038738) | | | | | |
| Hypoxia (PT: 10021143) | 6 | 15.04 (6.73-33.61) | 14.93 (77.89) | 3.90 (1.22) | 14.91 (6.67) |
| Pleural effusion (PT: 10035598) | 5 | 8.11 (3.36-19.54) | 8.06 (30.91) | 3.01 (0.70) | 8.05 (3.34) |
| Vascular disorders (SOC: 10047065) | | | | | |
| Hypotension (PT: 10021097) | 11 | 5.25 (2.89-9.52) | 5.19 (37.27) | 2.37 (1.10) | 5.19 (2.86) |
| Cardiac disorders (SOC: 10007541) | | | | | |
| Atrial fibrillation (PT: 10003658) | 7 | 6.74 (3.20-14.19) | 6.69 (33.87) | 2.74 (0.94) | 6.68 (3.17) |
| Psychiatric disorders (SOC: 10037175) | | | | | |
| Delirium (PT: 10012218) | 4 | 9.47 (3.54-25.32) | 9.43 (30.11) | 3.24 (0.51) | 9.42 (3.52) |
| Disorientation (PT: 10013395) | 3 | 9.97 (3.21-31.01) | 9.94 (24.09) | 3.31 (0.17) | 9.93 (3.19) |
| Renal and urinary disorders (SOC: 10038359) | | | | | |
| Renal impairment (PT: 10062237) | 6 | 5.63 (2.52-12.57) | 5.59 (22.64) | 2.48 (0.66) | 5.59 (2.50) |
| Blood and lymphatic system disorders (SOC: 10005329) | | | | | |
| Febrile neutropenia (PT: 10016288) | 5 | 5.54 (2.30-13.35) | 5.51 (18.46) | 2.46 (0.47) | 5.51 (2.28) |
| Metabolism and nutrition disorders (SOC: 10027433) | | | | | |
| Tumour lysis syndrome (PT: 10045170) | 3 | 23.08 (7.42-71.85) | 23.00 (62.95) | 4.52 (0.37) | 22.93 (7.37) |

The AE reports of epcoritamab taking 0.16 mg dose were included. **Abbreviations:** PT, preferred term; ROR, reporting odds ratio; CI, confidence interval; PRR, proportional reporting ratio; χ2, chi-squared; IC, information component; IC025, lower limit of 95% confidence interval of IC; EBGM, empirical Bayesian geometric mean; EBGM05, lower limit of 95% confidence interval of EBGM.

## Supplementary Table S14. Number and signal strength of epcoritamab-related signals at the PT level restricted to patients taking 48 mg dose of epcoritamab from the FAERS database.

| PT | Number | ROR (95% CI) | PRR (χ2) | IC (IC025) | EBGM (EBGM05) |
| --- | --- | --- | --- | --- | --- |
| Infections and infestations (SOC: 10021881) | | | | | |
| Sepsis (PT: 10040047) | 5 | 14.33 (5.91-34.78) | 14.04 (60.61) | 3.81 (0.95) | 14.03 (5.78) |
| Septic shock (PT: 10040070) | 3 | 19.92 (6.37-62.26) | 19.67 (53.16) | 4.30 (0.34) | 19.66 (6.29) |
| Cytomegalovirus infection reactivation (PT: 10058666) | 3 | 83.25 (26.60-260.53) | 82.16 (239.81) | 6.36 (0.49) | 81.91 (26.17) |
| Immune system disorders (SOC: 10021428) | | | | | |
| Cytokine release syndrome (PT: 10052015) | 10 | 64.71 (34.31-122.07) | 61.90 (598.24) | 5.95 (2.35) | 61.76 (32.74) |
| Blood and lymphatic system disorders (SOC: 10005329) | | | | | |
| Neutropenia (PT: 10029354) | 5 | 8.28 (3.41-20.08) | 8.12 (31.27) | 3.02 (0.70) | 8.11 (3.34) |
| Febrile neutropenia (PT: 10016288) | 4 | 15.16 (5.64-40.76) | 14.91 (51.95) | 3.90 (0.67) | 14.90 (5.54) |
| General disorders and administration site conditions (SOC: 10018065) | | | | | |
| Pyrexia (PT: 10037660) | 7 | 5.87 (2.77-12.46) | 5.72 (27.40) | 2.52 (0.81) | 5.72 (2.69) |
| Gastrointestinal disorders (SOC: 10017947) | | | | | |
| Abdominal pain (PT: 10000081) | 5 | 6.57 (2.71-15.95) | 6.45 (23.09) | 2.69 (0.56) | 6.45 (2.66) |
| Metabolism and nutrition disorders (SOC: 10027433) | | | | | |
| Dehydration (PT: 10012174) | 3 | 7.86 (2.52-24.57) | 7.77 (17.73) | 2.96 (0.07) | 7.77 (2.49) |

The AE reports of epcoritamab taking 48 mg dose were included. **Abbreviations:** PT, preferred term; ROR, reporting odds ratio; CI, confidence interval; PRR, proportional reporting ratio; χ2, chi-squared; IC, information component; IC025, lower limit of 95% confidence interval of IC; EBGM, empirical Bayesian geometric mean; EBGM05, lower limit of 95% confidence interval of EBGM.

## Supplementary Table S15. Number and signal strength of epcoritamab-related signals at the PT level based on reports occur within 1 week of medication from the FAERS database.

| PT | Number | ROR (95% CI) | PRR (χ2) | IC (IC025) | EBGM (EBGM05) |
| --- | --- | --- | --- | --- | --- |
| Immune system disorders (SOC: 10021428) | | | | | |
| Cytokine release syndrome (PT: 10052015) | 32 | 179.95 (124.41-260.30) | 159.72 (5013.03) | 7.31 (4.25) | 158.53 (109.60) |
| Infections and infestations (SOC: 10021881) | | | | | |
| Infection (PT: 10021789) | 6 | 8.11 (3.61-18.21) | 7.96 (36.59) | 2.99 (0.89) | 7.96 (3.54) |
| Sepsis (PT: 10040047) | 6 | 13.78 (6.14-30.96) | 13.51 (69.59) | 3.76 (1.17) | 13.50 (6.01) |
| Bacteraemia (PT: 10003997) | 3 | 54.07 (17.31-168.91) | 53.51 (154.22) | 5.74 (0.47) | 53.38 (17.09) |
| Investigations (SOC: 10022891) | | | | | |
| Neutrophil count decreased (PT: 10029366) | 6 | 25.91 (11.53-58.19) | 25.38 (140.46) | 4.66 (1.40) | 25.35 (11.29) |
| Platelet count decreased (PT: 10035528) | 5 | 9.66 (3.99-23.39) | 9.50 (38.10) | 3.25 (0.78) | 9.50 (3.92) |
| C-reactive protein increased (PT: 10006825) | 4 | 19.64 (7.32-52.72) | 19.38 (69.70) | 4.28 (0.75) | 19.36 (7.21) |
| General disorders and administration site conditions (SOC: 10018065) | | | | | |
| Pyrexia (PT: 10037660) | 10 | 6.76 (3.59-12.70) | 6.55 (47.30) | 2.71 (1.23) | 6.55 (3.49) |
| Chills (PT: 10008531) | 4 | 8.10 (3.02-21.73) | 8.00 (24.52) | 3.00 (0.43) | 7.99 (2.98) |
| Nervous system disorders (SOC: 10029205) | | | | | |
| Immune effector cell-associated neurotoxicity syndrome (PT: 10083347) | 7 | 103.13 (48.62-218.74) | 100.61 (687.23) | 6.65 (1.87) | 100.14 (47.21) |
| Vascular disorders (SOC: 10047065) | | | | | |
| Hypotension (PT: 10021097) | 7 | 9.16 (4.33-19.40) | 8.96 (49.61) | 3.16 (1.13) | 8.96 (4.23) |
| Gastrointestinal disorders (SOC: 10017947) | | | | | |
| Abdominal pain (PT: 10000081) | 5 | 5.25 (2.17-12.71) | 5.17 (16.88) | 2.37 (0.42) | 5.17 (2.14) |
| Renal and urinary disorders (SOC: 10038359) | | | | | |
| Renal impairment (PT: 10062237) | 3 | 7.66 (2.45-23.89) | 7.59 (17.17) | 2.92 (0.06) | 7.58 (2.43) |
| Respiratory, thoracic and mediastinal disorders (SOC: 10038738) | | | | | |
| Hypoxia (PT: 10021143) | 3 | 20.45 (6.55-63.83) | 20.24 (54.86) | 4.34 (0.35) | 20.23 (6.48) |

For epcoritamab, only the reports occur within 1 week of medication were included. **Abbreviations:** PT, preferred term; ROR, reporting odds ratio; CI, confidence interval; PRR, proportional reporting ratio; χ2, chi-squared; IC, information component; IC025, lower limit of 95% confidence interval of IC; EBGM, empirical Bayesian geometric mean; EBGM05, lower limit of 95% confidence interval of EBGM.

## Supplementary Table S16. Number and signal strength of epcoritamab-related signals at the PT level based on reports occur within 2-4 weeks of medication from the FAERS database.

| PT | Number | ROR (95% CI) | PRR (χ2) | IC (IC025) | EBGM (EBGM05) |
| --- | --- | --- | --- | --- | --- |
| Immune system disorders (SOC: 10021428) | | | | | |
| Cytokine release syndrome (PT: 10052015) | 55 | 306.09 (228.36-410.28) | 251.96 (13581.56) | 7.96 (5.10) | 248.74 (185.58) |
| General disorders and administration site conditions (SOC: 10018065) | | | | | |
| Pyrexia (PT: 10037660) | 17 | 10.71 (6.56-17.46) | 10.17 (141.30) | 3.35 (2.05) | 10.17 (6.23) |
| Nervous system disorders (SOC: 10029205) | | | | | |
| Immune effector cell-associated neurotoxicity syndrome (PT: 10083347) | 12 | 164.30 (92.05-293.25) | 157.98 (1857.17) | 7.29 (2.77) | 156.71 (87.80) |
| Infections and infestations (SOC: 10021881) | | | | | |
| Sepsis (PT: 10040047) | 4 | 8.32 (3.10-22.31) | 8.22 (25.40) | 3.04 (0.45) | 8.22 (3.06) |
| Staphylococcal infection (PT: 10058080) | 3 | 26.25 (8.41-81.91) | 26.01 (72.06) | 4.70 (0.39) | 25.97 (8.32) |
| Cytomegalovirus infection (PT: 10011831) | 3 | 39.34 (12.60-122.78) | 38.97 (110.78) | 5.28 (0.44) | 38.89 (12.46) |
| Investigations (SOC: 10022891) | | | | | |
| Neutrophil count decreased (PT: 10029366) | 6 | 23.61 (10.52-52.98) | 23.17 (127.22) | 4.53 (1.37) | 23.14 (10.31) |
| Platelet count decreased (PT: 10035528) | 4 | 7.02 (2.62-18.82) | 6.94 (20.37) | 2.79 (0.36) | 6.94 (2.59) |
| Vascular disorders (SOC: 10047065) | | | | | |
| Hypotension (PT: 10021097) | 6 | 7.13 (3.18-15.99) | 7.01 (30.99) | 2.81 (0.81) | 7.01 (3.12) |
| Cardiac disorders (SOC: 10007541) | | | | | |
| Tachycardia (PT: 10043071) | 3 | 7.74 (2.48-24.15) | 7.68 (17.44) | 2.94 (0.07) | 7.68 (2.46) |

For epcoritamab, only the reports occur within 2-4 weeks of medication were included. **Abbreviations:** PT, preferred term; ROR, reporting odds ratio; CI, confidence interval; PRR, proportional reporting ratio; χ2, chi-squared; IC, information component; IC025, lower limit of 95% confidence interval of IC; EBGM, empirical Bayesian geometric mean; EBGM05, lower limit of 95% confidence interval of EBGM.

## Supplementary Table S17. Number and signal strength of epcoritamab-related signals at the PT level based on reports occur after 4 weeks of medication from the FAERS database.

| PT | Number | ROR (95% CI) | PRR (χ2) | IC (IC025) | EBGM (EBGM05) |
| --- | --- | --- | --- | --- | --- |
| Infections and infestations (SOC: 10021881) | | | | | |
| Cytomegalovirus infection (PT: 10011831) | 7 | 156.10 (73.24-332.69) | 150.33 (1033.74) | 7.23 (1.89) | 149.63 (70.21) |
| Urinary tract infection (PT: 10046571) | 4 | 7.16 (2.66-19.28) | 7.03 (20.74) | 2.81 (0.36) | 7.03 (2.61) |
| Pneumonia cytomegaloviral (PT: 10035676) | 3 | 521.30 (165.11-1645.90) | 513.00 (1508.84) | 8.98 (0.52) | 504.91 (159.92) |
| Pneumonia bacterial (PT: 10060946) | 3 | 76.99 (24.57-241.22) | 75.77 (220.89) | 6.24 (0.48) | 75.60 (24.13) |
| Cytomegalovirus infection reactivation (PT: 10058666) | 3 | 100.80 (32.16-315.95) | 99.20 (290.79) | 6.63 (0.50) | 98.90 (31.55) |
| Progressive multifocal leukoencephalopathy (PT: 10036807) | 3 | 130.84 (41.72-410.33) | 128.77 (378.84) | 7.00 (0.50) | 128.25 (40.89) |
| Sepsis (PT: 10040047) | 3 | 10.32 (3.30-32.29) | 10.17 (24.83) | 3.35 (0.17) | 10.16 (3.25) |
| Pneumocystis jirovecii pneumonia (PT: 10073755) | 3 | 79.76 (25.45-249.92) | 78.50 (229.03) | 6.29 (0.49) | 78.31 (24.99) |
| Blood and lymphatic system disorders (SOC: 10005329) | | | | | |
| Febrile neutropenia (PT: 10016288) | 6 | 27.87 (12.36-62.88) | 27.02 (150.36) | 4.75 (1.41) | 26.99 (11.97) |
| Neutropenia (PT: 10029354) | 4 | 7.99 (2.97-21.51) | 7.84 (23.93) | 2.97 (0.42) | 7.84 (2.91) |
| Immune system disorders (SOC: 10021428) | | | | | |
| Cytokine release syndrome (PT: 10052015) | 4 | 30.48 (11.32-82.13) | 29.86 (111.54) | 4.90 (0.83) | 29.83 (11.07) |

For epcoritamab, only the reports occur after 4 weeks of medication were included. **Abbreviations:** PT, preferred term; ROR, reporting odds ratio; CI, confidence interval; PRR, proportional reporting ratio; χ2, chi-squared; IC, information component; IC025, lower limit of 95% confidence interval of IC; EBGM, empirical Bayesian geometric mean; EBGM05, lower limit of 95% confidence interval of EBGM.
